# Loss of Fibroblast-Dependent Androgen Receptor Activation in Prostate Cancer Cells is Involved in the Mechanism of Acquired Resistance to Castration

**DOI:** 10.3390/jcm8091379

**Published:** 2019-09-03

**Authors:** Kenichiro Ishii, Izumi Matsuoka, Takeshi Sasaki, Kohei Nishikawa, Hideki Kanda, Hiroshi Imai, Yoshifumi Hirokawa, Kazuhiro Iguchi, Kiminobu Arima, Yoshiki Sugimura

**Affiliations:** 1 Department of Nephro-Urologic Surgery and Andrology, Mie University Graduate School of Medicine, Tsu, Mie 514-8507, Japan (I.M.) (T.S.) (K.N.) (H.K.) (K.A.) (Y.S.); 2 Department of Oncologic Pathology, Mie University Graduate School of Medicine, Tsu, Mie 514-8507, Japan; 3 Pathology Division, Mie University Hospital, Tsu, Mie 514-8507, Japan; 4 Laboratory of Community Pharmacy, Gifu Pharmaceutical University, Gifu, Gifu 501-1196, Japan

**Keywords:** prostate cancer, androgen deprivation therapy, androgen sensitivity, androgen receptor dependency, fibroblast-dependent androgen receptor activation

## Abstract

Loss of androgen receptor (AR) dependency in prostate cancer (PCa) cells is associated with progression to castration-resistant prostate cancer (CRPC). The tumor stroma is enriched in fibroblasts that secrete AR-activating factors. To investigate the roles of fibroblasts in AR activation under androgen deprivation, we used three sublines of androgen-sensitive LNCaP cells (E9 and F10 cells: low androgen sensitivity; and AIDL cells: androgen insensitivity) and original fibroblasts derived from patients with PCa. We performed in vivo experiments using three sublines of LNCaP cells and original fibroblasts to form homotypic tumors. The volume of tumors derived from E9 cells plus fibroblasts was reduced following androgen deprivation therapy (ADT), whereas that of F10 or AIDL cells plus fibroblasts was increased even after ADT. In tumors derived from E9 cells plus fibroblasts, serum prostate-specific antigen (PSA) decreased rapidly after ADT, but was still detectable. In contrast, serum PSA was increased even in F10 cells inoculated alone. In indirect cocultures with fibroblasts, PSA production was increased in E9 cells. Epidermal growth factor treatment stimulated Akt and p44/42 mitogen-activated protein kinase phosphorylation in E9 cells. Notably, AR splice variant 7 was detected in F10 cells. Overall, we found that fibroblast-secreted AR-activating factors modulated AR signaling in E9 cells after ADT and loss of fibroblast-dependent AR activation in F10 cells may be responsible for CRPC progression.

## 1. Introduction

The number of men diagnosed with prostate cancer (PCa) is increasing worldwide [1]. Most patients with early-stage PCa can be treated with therapies such as radical prostatectomy or irradiation. In contrast, patients with advanced PCa are treated with androgen deprivation therapy (ADT), the standard systemic therapy. Although ADT induces temporary remission, the majority of patients (approximately 60%) eventually develop and progress to castration-resistant prostate cancer (CRPC), which is associated with a high mortality rate [2,3]. In the development and progression of CRPC, a decrease or loss of androgen sensitivity in PCa cells often occurs. Low androgen sensitivity of PCa cells is associated with a more malignant phenotype and is difficult to cure. Changes in the androgen sensitivity of PCa cells are often caused artificially as negative effects of ADT and by spontaneously arising variants of androgen receptor (AR) even before ADT is started [4,5]. 

ADT for patients with advanced PCa aims to decrease the concentration of circulating androgen and block AR signaling in PCa cells [6]. Well-differentiated PCa cells are generally androgen and AR dependent, i.e., AR signaling regulates cell cycle progression and differentiation. Loss of AR signaling after ADT triggers AR-independent outgrowth, generating poorly differentiated uncontrollable PCa cells [7]. To prevent the development and progression of CRPC, we hypothesize that preservation of AR signaling after ADT is essential. Nelson et al. described four molecular-state frameworks for activation of AR signaling after ADT as follows: state 1, endocrine androgen and AR dependent; state 2, intracrine androgen and AR dependent; state 3, androgen independent and AR dependent; and state 4, androgen and AR independent [8]. Several molecular mechanisms responsible for changes in the AR dependency of PCa cells have been suggested, e.g., androgen-independent activation of AR signaling by mutations in the AR or altered levels of coactivators, and activation of alternative growth factor pathways [4,5]. In patients with CRPC, a number of growth factors and cytokines contribute to malignancy of PCa cells through activation of AR signaling in an androgen-independent manner, which is often called the “outlaw pathway” [8]. Previous studies have suggested that epidermal growth factor (EGF), fibroblast growth factor (FGF)-7 (also known as keratinocyte growth factor (KGF)), insulin-like growth factor (IGF)-1, and interleukin (IL)-6 can activate AR signaling in the absence of androgen [9,10,11] via various signaling pathways, including Akt, signal transducer and activator of transcription (STAT) 3, and p44/42 mitogen-activated protein kinase (MAPK) pathways [12]. In the tumor microenvironment, tumor stroma surrounding PCa cells is enriched in fibroblasts that secrete AR-activating factors, such as EGF, FGF-7/KGF, IGF-1, and IL-6 [13,14].

Most fibroblasts do not express AR and can survive in the absence of androgen [15,16,17]. Several studies have reported that androgen-independent interactions between PCa cells and fibroblasts determine how PCa cells respond to ADT [18,19]. In androgen-insensitive PCa cells, we demonstrated that fibroblast-derived FGF-7/KGF may bypass the functionally inactive AR and may promote cell proliferation after ADT [19]. In androgen-sensitive PCa cells, however, we have recently reported that fibroblast-derived EGF, IGF-1, and IL-6 can activate AR signaling, leading to preservation of AR signaling after ADT [17]. Thus, we hypothesize that fibroblast-dependent AR activation may preserve AR signaling after ADT and may play a critical role in the prevention of CRPC development and progression. Clinically, PCa is a heterogeneous disease with various biological behaviors, such as androgen sensitivity and response to ADT. To investigate the relationship between fibroblast-dependent AR activation and androgen sensitivity in PCa cells, well-established PCa cell lines with a variety of androgen sensitivities are strongly required. 

LNCaP human PCa cell lines are androgen-sensitive PCa cell lines that are useful for investigating the molecular mechanisms responsible for changes in the androgen sensitivity and AR dependency of PCa cells. Notably, LNCaP cells are a heterogeneous cell population containing various clones with naturally occurring differences in androgen sensitivity caused by spontaneously arising changes [20,21]. Accordingly, we generated two sublines of LNCaP cells (E9 and F10) showing low androgen sensitivity by using a limiting dilution method with regular culture conditions [22,23]. In addition, we established androgen-insensitive AIDL cells from parental LNCaP cells by continuous passaging under hormone-depleted conditions [24]. The parental LNCaP cell line and its derivative sublines (E9, F10, and AIDL) expressed similar levels of AR protein, and AR-dependent secretion of prostate-specific antigen (PSA) was detected in LNCaP and E9 cells [14]. As compared with parental LNCaP cells, we have previously reported that combination of E9 or AIDL cells with embryonic rat urogenital sinus mesenchyme promoted tumor progression in vivo even under androgen ablation [19]. Comparing the characteristic features of paternal LNCaP cells and its sublines allows us to investigate the molecular mechanisms responsible for changes in the androgen sensitivity and AR dependency of PCa cells. 

In this study, we defined “androgen sensitivity” in PCa cells as the degree of androgen-dependent AR activation in vitro. In contrast, we defined “AR dependency” in PCa cells as the degree of androgen- and growth factor/cytokine-dependent AR activation. The objective of this study was to investigate the role of fibroblast-dependent AR activation in the tumorigenesis of three LNCaP sublines differing in androgen sensitivity under androgen deprivation conditions. 

## 2. Materials and Methods

### 2.1. Materials

Dihydrotestosterone (DHT) and anti-androgen bicalutamide were purchased from Sigma-Aldrich Co., LLC. (St. Louis, MO, USA). Recombinant human EGF, FGF-2, FGF-7, FGF-10, hepatocyte growth factor (HGF), IGF-1, transforming growth factor (TGF) β1, vascular endothelial growth factor (VEGF), and IL-6 were purchased from PeproTech, Inc. (Rocky Hill, NJ, USA). Rabbit polyclonal anti-PSA and mouse monoclonal anti-neuron-specific enolase (NSE; BBS/NC/V1-H14) antibodies were purchased from Dako Cytomation (Copenhagen, Denmark). Rabbit polyclonal anti-AR (N-20) and anti-EGFR (1005) antibodies were purchased from Santa Cruz Biotechnology (Santa Cruz, CA, USA). Rabbit polyclonal anti-CD31 and anti-Ki-67 and rabbit monoclonal anti-AR splice variant 7 (AR-V7; EPR15656) antibodies were purchased from Abcam Inc. (Cambridge, MA, USA). Rabbit monoclonal anti-phospho-STAT3 (Tyr705) (D3A7), anti-STAT3 (D3Z2G), anti-phospho-Akt (Ser473) (D9E), and anti-Akt (pan) (C67E7) antibodies were purchased from Cell Signaling Technology, Inc. (Beverly, MA, USA). Rabbit polyclonal anti-p44/42 MAPK and mouse monoclonal anti-phospho-p44/42 MAPK (Thr202/Tyr204) (E10) antibodies were purchased from Cell Signaling Technology, Inc. Mouse monoclonal anti-β-actin (AC-15) antibodies were purchased from Sigma-Aldrich Co., LLC. 

### 2.2. Cell Culture

The androgen-sensitive, AR-positive human PCa cell lines LNCaP and 22Rv1 were obtained from the American Type Culture Collection (Manassas, VA, USA). Both LNCaP and 22Rv1 cells were authenticated by the short tandem repeat-PCR method and were cultured in phenol red (+) RPMI-1640 supplemented with 10% fetal bovine serum (FBS; Sigma-Aldrich Co., LLC.). E9 and F10 cells (showing low sensitivity to androgen) were obtained from the parental androgen-sensitive LNCaP cells using a limiting dilution method under regular culture conditions [22,23]. In contrast, androgen-insensitive AIDL cells were established from LNCaP cells by continuous passaging under hormone-depleted conditions [24]. AIDL cells were cultured in phenol red (-) RPMI-1640 supplemented with 10% charcoal-stripped (CS)-FBS. The androgen sensitivity of parental LNCaP, E9, F10, and AIDL cells was confirmed by changes in *KLK3* (PSA) mRNA expression in cell cultures treated with synthetic androgen R1881 [19]. The nontumorigenic human prostate epithelial cell line BPH-1 was obtained from Dr. Simon W. Hayward (Northshore University HealthSystem, Chicago, IL, USA) and was cultured in phenol red (+) RPMI-1640 supplemented with 10% FBS.

Commercially available human prostate stromal cells (PrSC) were purchased from Lonza Group Ltd. (Basel, Switzerland). pcPrFs (pcPrF-M5, pcPrF-M6, and pcPrF-M7) were primary cultured from PCa specimens collected from biopsies of patients with advanced PCa [17]. PrSC and pcPrFs were cultured in medium prepared from an SCBM Bullet Kit (Lonza Group Ltd.). The four fibroblast lines (PrSC, pcPrF-M5, pcPrF-M6, and pcPrF-M7) do not express AR protein and do not respond to DHT stimulation on cell proliferation as previously reported [17].

### 2.3. Indirect Coculture of Prostate Cancer Cell Lines (E9, F10, and AIDL Cells) with Fibroblasts

E9, F10, and AIDL cells were co-cultured with each of the four fibroblast lines (PrSC, pcPrF-M5, pcPrF-M6, and pcPrF-M7) in six-well plates using cell culture inserts (BD Falcon, Franklin Lakes, NJ, USA) as previously described [17]. E9, F10, and AIDL cells (4 × 10^4^ cells/well) were seeded into six-well plates in their respective recommended medium, whereas fibroblasts (PrSC, pcPrF-M5, pcPrF-M6, and pcPrF-M7; 2 × 10^4^ cells/well) were seeded in SCBM media into cell culture inserts for 2 days. The culture medium for PCa cells and fibroblasts was replaced with phenol red (-) RPMI-1640 supplemented with 1% CS-FBS containing DHT (0.1 nM), and the inserts with fibroblasts were then placed into six-well plates for an additional 4 days. DHT concentrations in the incubation medium were chosen based on previous studies of tissue DHT levels in recurrent PCa [25].

### 2.4. Stimulation of Cell Growth by Treatment with Growth Factors and Cytokines

Examination of the effects of growth factor and cytokine stimulation was performed as previously described [17], with minor modifications. E9 (5 × 10^3^ cells/well), F10 (4 × 10^3^ cells/well), and AIDL cells (6 × 10^3^ cells/well) were cultured in phenol red (+) RPMI-1640 supplemented with 10% FBS for 2 days, and the culture medium was then replaced with phenol red (-) RPMI-1640 supplemented with 1% CS-FBS containing DHT (0.1 nM). One day later, the culture medium was replaced with phenol red (-) RPMI-1640 containing 10 ng/mL each of recombinant EGF, FGF-2, FGF-7, FGF-10, HGF, IGF-1, TGFβ1, VEGF, and IL-6. Cells were then incubated for 4 days before analysis.

### 2.5. Enzyme-Linked Immunosorbent Assay

Serum PSA levels in mice were assayed using a PSA Enzyme Immunoassay test kit (Hope Laboratories, Belmont, CA, USA). 

### 2.6. Preparation of Cell Lysates

LNCaP, E9, F10, AIDL, and 22Rv1 cells were harvested by scraping, and whole cell lysates were prepared as previously described [14]. Briefly, the cell surface was washed with ice-cold phosphate-buffered saline and then lysed with CelLytic (Sigma-Aldrich Co., LLC.) containing 1% Nonidet P-40, 10 mM 4-(2-aminoethyl) benzensulfonyl fluoride, 0.8 mM aprotinin, 50 mM bestatin, 15 mM E-64, 20 mM leupeptin, and 10 mM pepstatin. After 60 min on ice, the lysates were centrifuged at 10,000× *g* for 10 min, and the supernatants were collected. The protein concentration was measured using a NanoDrop 2000 (Thermo Fisher Scientific Inc., Waltham, MA, USA).

### 2.7. Western Blot Analyses

Extracted proteins were separated by gel electrophoresis and transferred to Immobilon polyvinylidene difluoride membranes following our previously reported protocol [26]. Anti-AR, anti-PSA, anti-NSE, anti-phospho-STAT3, anti-STAT3, anti-phospho-Akt, anti-Akt, anti-phospho-p44/42 MAPK, anti-p44/42 MAPK, and anti-β-actin antibodies were used at dilutions of 1:2500, 1:5000, 1:5000, 1:2000, 1:2000, 1:1000, 1:1000, 1:2000, 1:1000, and 1:5000, respectively. Specific protein bands were assessed with a LAS-4000 Mini (Fuji Photo Film, Tokyo, Japan) using SuperSignal West Pico Chemiluminescent Substrate (Thermo Fisher Scientific Inc.). 

### 2.8. Animal Studies

All animals were maintained in a specific pathogen-free environment. Mie University’s Committee on Animal Investigations approved the experimental protocol. Male athymic nude mice (BALB/c, nu/nu, 6–8 weeks old) were purchased from CLEA Japan, Inc. (Tokyo, Japan) and used for all experiments. 

### 2.9. In Vivo Xenograft Model

Examination of the effects of ADT on tumorigenesis of E9, F10, and AIDL cells was performed as previously described [17]. Subconfluent cultures of E9, F10, AIDL, and pcPrF-M5 cells were trypsinized and counted. Xenografts without pcPrF-M5 cells contained 5 × 10^5^ PCa cells. Xenografts with pcPrF-M5 cells were prepared by mixing 2.5 × 10^5^ PCa cells and 2.5 × 10^5^ pcPrF-M5 cells in suspension. Pelleted cells were resuspended in 50 µL neutralized type I rat tail collagen gels and then grafted beneath the renal capsule of male athymic mice (6–8 weeks old). In total, 1 × 10^6^ PCa cells were grafted in each mouse. For the androgen deprivation experiments, mice were randomized on day 14 after transplantation. Mice treated with ADT were castrated and orally administered a bicalutamide (5 mg/kg/day) suspension with 0.5% carboxymethylcellulose in a 5-days-on/2-days-off schedule through a 22-gauge gavage needle; the control group underwent sham operation and received the diluent. Mice were killed on days 14 and 21 after ADT, and tumor weights and serum PSA levels were then measured. The tumor volume was determined by direct measurement with calipers (volume = long axis × short axis ×short axis × 0.5236), as previously described [27]. 

### 2.10. Histopathology and Immunohistochemistry

For histopathological analysis, tumors were fixed in 10% neutral buffered formalin and embedded in paraffin. General tissue morphology was visualized by standard hematoxylin and eosin staining. Next, immunohistochemical staining was performed with an ImmPRESS Reagent Kit (Vector Laboratories, Inc., Burlingame, CA, USA). Antigen retrieval was performed using 10 mM sodium citrate buffer (pH 6.0) for AR, NSE, Ki-67, and CD31. Antigen Unmasking Solution (Vector Laboratories, Inc.) was used for PSA. The antigen-antibody reaction was visualized using 3′,3-diaminobenzidine tertahydrochloride as a substrate. Sections were counterstained with hematoxylin and examined by light microscopy. 

Cell proliferation in tumors was determined by the percentage of Ki67-positive nuclei in 10 different areas at 400× magnification from each tissue specimen. A ‘microvessel’ was defined as mouse-specific CD31-positive endothelial cells that formed a vascular lumen. The number of microvessels was counted in 10 different areas at 400× magnification from each tissue specimen, as previously described [27]. The results were independently reviewed by 2 blinded investigators.

### 2.11. Statistical Analysis

Results are expressed as means ± standard deviations. Differences between the two groups were determined using Student’s *t*-tests. Results with *p* values of less than 0.05 were considered statistically significant.

## 3. Results

### 3.1. Effects of ADT on Tumor Growth and Serum PSA Kinetics of Xenograft Derived from Co-Inoculation of E9, F10, and AIDL Cells with pcPrF-M5 Cells In Vivo

**Figure 1:**
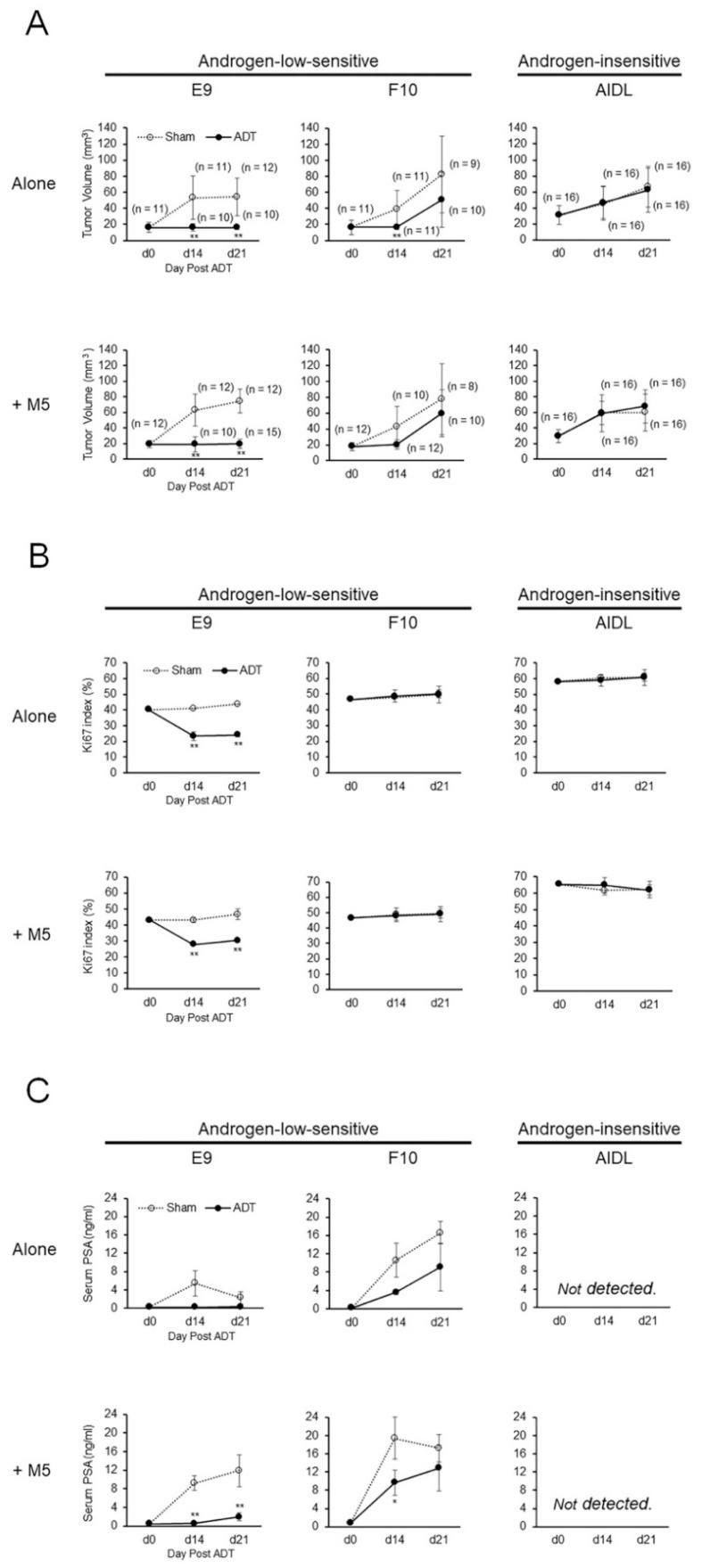
Effects of androgen deprivation therapy (ADT) on tumor growth and serum PSA kinetics of xenografts derived from co-inoculation of LNCaP sublines with pcPrF-M5 cells in vivo. Changes in tumor volume (**A**), Ki67 index (**B**), and serum PSA (**C**) of xenografts were compared in untreated (sham-operated) or ADT-treated mice inoculated with PCa cells with or without M5 cells on days 0, 14, and 21 after ADT. ** *P* < 0.01 versus sham-operated control. ADT, androgen deprivation therapy; PSA, prostate-specific antigen; M5, pcPrF-M5.

Regardless of the presence of pcPrF-M5 cells, tumor volumes in mice inoculated with E9 cells rapidly decreased following ADT (Figure 1A) and were not altered between days 14 and 21 after ADT, i.e., the effects of ADT on E9 tumors were maintained until at least day 21 after ADT. In contrast, in the presence or absence of pcPrF-M5 cells, tumor volumes in mice inoculated with F10 cells temporally decreased following ADT, but increased between days 14 and 21 after ADT (Figure 1A). Moreover, in the presence or absence of pcPrF-M5 cells, tumor volumes in mice inoculated with AIDL cells gradually increased following ADT, similar to the results in the sham group (Figure 1A). 

Ki-67 labeling indices in mice inoculated with E9 cells, with or without pcPrF-M5 cells, rapidly decreased following ADT (Figure 1B) and were not significantly increased between days 14 and 21 after ADT; that is, the effects of ADT on E9 tumors were maintained until at least day 21 after ADT. In contrast, Ki-67 labeling indices in mice inoculated with F10 or AIDL cells, with or without pcPrF-M5 cells, were not altered following ADT as compared with that in the sham group (Figure 1B).

Serum PSA titers in mice inoculated with E9 cells alone rapidly decreased after ADT (Figure 1C). Importantly, those in mice inoculated with E9 cells plus pcPrF-M5 cells rapidly decreased following ADT and became detectable on day 21 after ADT. Serum PSA titers in mice inoculated with F10 cells, with or without pcPrF-M5 cells, increased gradually (Figure 1C), whereas those in mice inoculated with AIDL cells, with or without pcPrF-M5 cells, were not detected (Figure 1C). 

E9-derived tumors, with or without pcPrF-M5 cells, grown in mice treated with ADT showed reduced tumorigenesis as compared with those in untreated (sham-operated) mice (Figure S1). In contrast, both F10- and AIDL-derived tumors, with or without pcPrF-M5 cells, grown in mice treated with ADT showed no changes in tumorigenesis as compared with those in untreated mice (Figures S2 and S3). Additionally, AR and PSA proteins were expressed in both E9- and F10-derived tumors, with or without pcPrF-M5 cells, on day 21 after ADT, although serum PSA levels were very low in mice inoculated with E9 cells alone (Figures S1 and S2). Moreover, AR protein was expressed in AIDL-derived tumors, with or without pcPrF-M5 cells, on day 21 after ADT, whereas PSA protein was not detected because of mutated AR in AIDL cells (Figure S3). NSE staining was diffuse among ADT-treated and untreated hosts on day 21 after ADT (Figures S1–S3). Microvessel density (MVD) in all tumors, regardless of the presence of pcPrF-M5 cells, was not altered among ADT-treated and untreated hosts on days 14 and 21 after ADT (Tables S1–S3). 

### 3.2. Effects of Indirect Coculture with Fibroblasts on E9, F10, and AIDL Cells In Vitro

**Figure 2:**
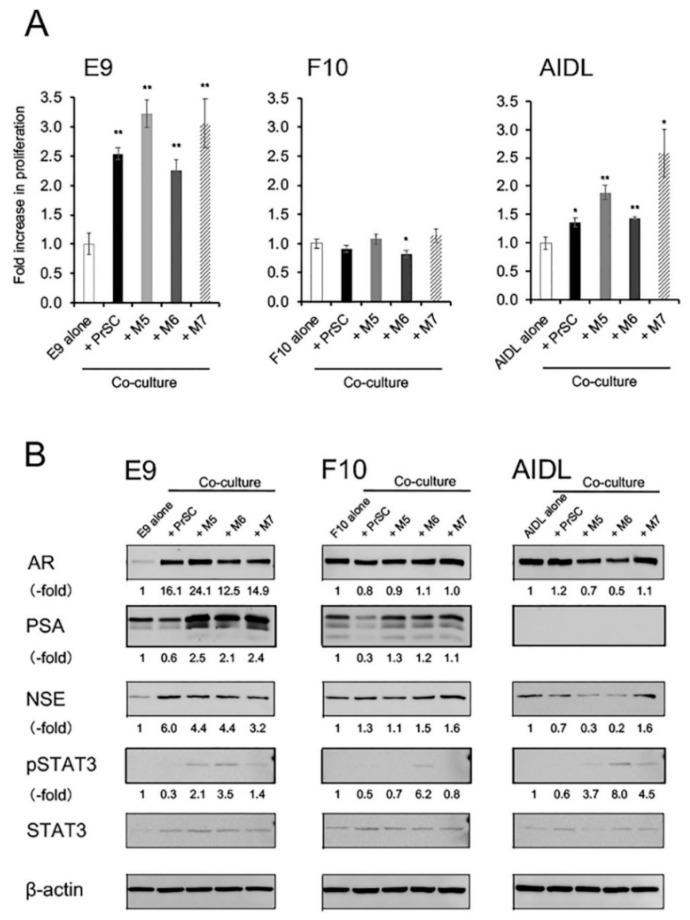
Effects of indirect coculture with fibroblasts on cell proliferation and PSA expression in LNCaP sublines in vitro. (**A** and **B**) LNCaP sublines were co-cultured with fibroblasts using cell culture inserts for 4 days in phenol red (−) RPMI-1640 with 1% CS-FBS containing DHT (0.1 nM). (**A**) Cell proliferation. * *P* < 0.05, ** *P* < 0.01 versus LNCaP sublines alone. (**B**) Cell lysates from co-cultures were subjected to western blotting and probed with antibodies against each protein. Protein levels were compared using actin expression as a loading control. AR, androgen receptor; DHT, dihydrotestosterone; NSE, neuron-specific enolase; PSA, prostate-specific antigen; M5, pcPrF-M5; M6, pcPrF-M6; M7, pcPrF-M7.

Cell proliferation of E9 and AIDL cells was significantly increased when cells were cocultured with PrSCs or pcPrFs, whereas that of F10 cells was not affected (Figure 2A). Expression levels of PSA proteins were increased in E9 cells cocultured with pcPrFs but not PrSC and were not affected in F10 cells (Figure 2B). In contrast, expression levels of PSA proteins were decreased in F10 cells cocultured with PrSC but not pcPrFs. PSA protein expression was not detected in AIDL cells cocultured with PrSC or pcPrFs (Figure 2B). NSE protein expression and STAT3 phosphorylation were increased in E9 cells cocultured with PrSC or pcPrFs (Figure 2B). NSE proteins were not changed in F10 cells cocultured with PrSC or pcPrFs, whereas STAT3 phosphorylation was increased in F10 cells cocultured with pcPrF-M6 cells (Figure 2B). NSE protein expression was decreased in AIDL cells cocultured with pcPrFs but not PrSC, whereas phosphorylation of STAT3 was increased in AIDL cells cocultured with pcPrFs (Figure 2B). 

### 3.3. Effects of Growth Factors and Cytokines on E9, F10, and AIDL Cells In Vitro

**Figure 3:**
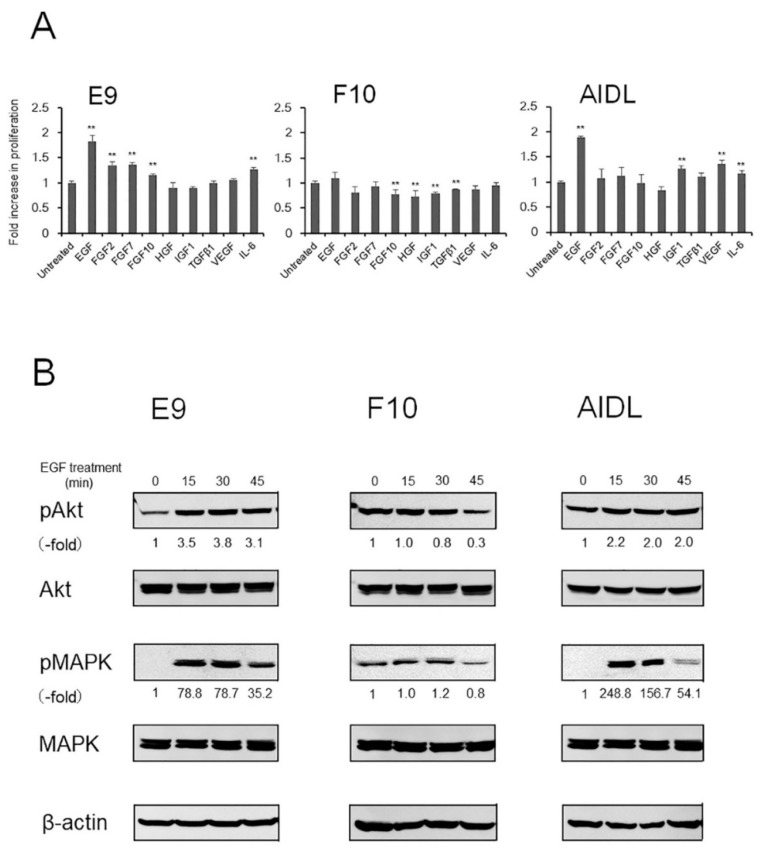
Effects of growth factors and cytokines on cell proliferation and cellular signaling in LNCaP sublines in vitro. (**A** and **B**) LNCaP sublines were treated with 10 ng/mL of each growth factor and cytokine for 4 days in phenol red (−) RPMI-1640 with 1% CS-FBS containing DHT (0.1 nM). (**A**) Cell proliferation. ** *P* < 0.01 versus untreated control. (**B**) Cell lysates from cultures of LNCaP sublines were subjected to western blotting and probed with antibodies against each target protein. Protein levels were compared with actin expression as a loading control. DHT, dihydrotestosterone.

Cell proliferation of E9 and AIDL cells was significantly increased by treatment with growth factors and cytokines, such as EGF and IL-6, whereas that of F10 cells was significantly decreased by treatment with FGF-10, HGF, IGF-1, and TGFβ1 (Figure 3A). Phosphorylation of Akt and p44/42 MAPK in E9 and AIDL cells was strongly increased by treatment with EGF, whereas that in F10 cells was not affected (Figure 3B). Phosphorylation of Akt and p44/42 MAPK in E9, F10, and AIDL cells was not affected by treatment with HGF (data not shown). EGFR protein was detectable in all PCa cells, including LNCaP, E9, F10, AIDL cells, whereas expression of EGFR protein in AIDL cells was considerably higher than that in E9 and F10 cells (Figure S4). Detection of EGFR protein in BPH-1 cells was used as a positive control for anti-EGFR antibodies. In addition, PSA secretion from E9 cells was significantly increased by treatment with EGF but not HGF, whereas that from F10 cells was significantly decreased by treatment with both EGF and HGF (Figure S5). Notably, PSA secretion from AIDL cells was not detected.

**Figure 4:**
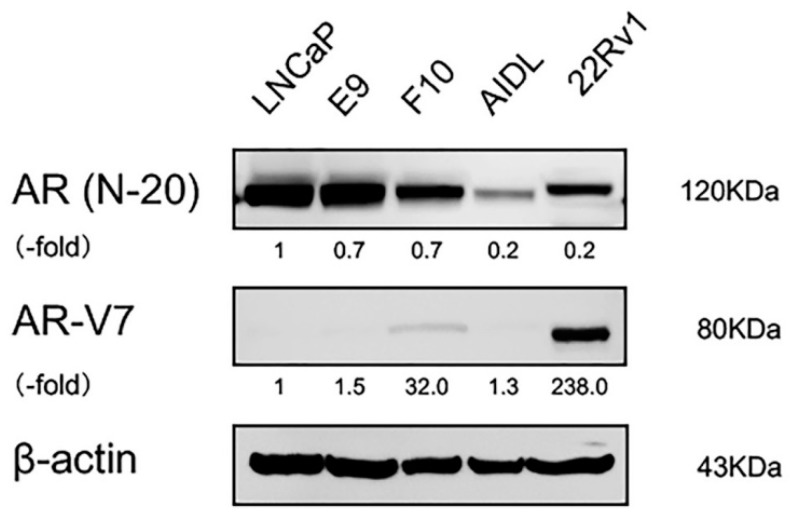
Expression of AR-V7 protein in human PCa cell lines. Cell lysates from growing cultures of parental LNCaP cells, LNCaP sublines (E9, F10, and AIDL cells), and 22Rv1 cells were subjected to western blotting and probed with antibodies against each protein. Protein levels were compared using actin as a loading control. 22Rv1 cells were used as a positive control for detection of AR-V7 protein. AR, androgen receptor; AR-V7, androgen receptor splice variant 7.

Full-length AR protein was detectable in all PCa cells, including LNCaP, E9, F10, AIDL, and 22Rv1 cells, whereas AR-V7 protein was detectable only in F10 cells (Figure 4). Detection of AR-V7 protein in 22Rv1 cells was used as a positive control for anti-AR-V7 antibodies.

## 4. Discussion

The reduced AR dependency of PCa cells is an important clinical development because of its association with progression to CRPC. In this study, we found that fibroblast-secreted AR-activating factors preserved AR signaling in E9 cells after ADT, indicating that these PCa cells could be controlled by ADT. In contrast, loss of fibroblast-dependent AR activation in F10 cells may be responsible for the development and progression of CRPC. 

Development and progression of CRPC after ADT are mediated by multiple molecular mechanisms, classified as adaptation to a low-concentration androgen environment caused by ADT, or clonal selection [28,29,30,31]. Androgen-insensitive PCa cells can be generated by adaptation of androgen-sensitive PCa cells to a low androgen environment. In contrast, ADT results in the expansion of androgen-insensitive PCa cells, which coexist with androgen-sensitive PCa cell populations in PCa tissue, i.e., clonal selection of androgen-insensitive PCa cells [28]. PCa tissue consists of heterogeneous cell populations. Tumor heterogeneity is reflected in the increased subclonal populations in PCa tissue [32]. These subclones may interact in complex ways with each other or with the surrounding microenvironment. Thus, we hypothesize that tumor heterogeneity in PCa tissue is an extremely important phenomenon not only for understanding tumor progression but also for developing truly personalized treatment regimens for patients with PCa. 

To compare the biochemical characteristics of androgen-sensitive and -insensitive PCa cells, we generated three sublines from androgen-sensitive AR-positive LNCaP cells: E9 and F10 cells (showing low androgen sensitivity) and AIDL cells (showing androgen insensitivity) [22,23,24]. E9 cells are less sensitive to androgen-related responses, such as growth stimulation and PSA production, than parental LNCaP cells [22]. Moreover, E9 cells have a more aggressive tumorigenic phenotype than parental LNCaP cells in vivo. We have previously investigated the mechanisms underlying the low androgen sensitivity of E9 cells and found that decreased phosphorylation of Akt was associated with low androgen sensitivity of E9 cells [33]. The Akt and p44/42 MAPK pathways are known to be associated with the regulation of androgen responses [34,35]. We also demonstrated that PSA production was significantly decreased in parental LNCaP cells when Akt phosphorylation was suppressed by phosphatidylinositol 3-kinase or Akt inhibitors [33]. Thus, E9 cells may be a useful model to reflect high-grade Gleason tumors with low phosphorylation of Akt. Similar to E9 cells, F10 cells are also less sensitive to androgen-related responses than parental LNCaP cells and have a more aggressive tumorigenic phenotype than parental LNCaP cells in vivo [23]. Interestingly, F10 cells can survive under low-pH/low-nutrient conditions, whereas parental LNCaP cells show significant cell death under such conditions. The intratumor environment is characterized by low-pH, low-nutrient, and chronic hypoxic conditions owing to poor vascular development [36,37]. Thus, we suggest that F10 cells may be a useful model to determine the mechanisms underlying their adaptation to a low-pH/low-nutrient environment. In contrast to E9 and F10 cells, AIDL cells are insensitive to androgen-related responses [24]. Parental LNCaP cells harbor an AR mutation at codon 877 (T877A). In addition to the T877A mutation, we found that AIDL cells harbored a point mutation at codon 741 (W741C), suggesting that the T877A/W741C double mutation may be responsible for the androgen insensitivity of AIDL cells [38]. Thus, AIDL cells may be a useful model to investigate the mechanisms underlying the mutated AR in PCa cells. 

In PCa, the tumor microenvironment is highly complex and heterogeneous and is composed of carcinoma-associated fibroblasts (CAFs) as well as epithelial cancer cells that infiltrate into the surrounding tumor stroma, referred to as the reactive stroma [39]. This heterogeneous stromal component of PCa tissues contains multiple populations of fibroblasts that are associated with tumorigenesis [40,41]. CAFs contribute to the malignancy of PCa cells by enhancing the proliferation and invasion of cancer cells and promoting angiogenesis in tumors [42]. Thus, inhibition of CAF generation and function in PCa tissue could be a new target for controlling primary cancer progression. Importantly, most fibroblasts in the prostate stroma are negative for AR [15,16,17], and the phenotypes of human PCa fibroblasts are strongly heterogeneous [13]. CAFs secrete abundant growth factors and cytokines, which enhance the proliferation of PCa cells. However, the proliferation of PCa cells is regulated by AR signaling, suggesting that stromal paracrine factors derived from CAFs can activate AR signaling in PCa cells. In patients with CRPC, PCa cells can grow in the absence of androgen, indicating that AR signaling in PCa cells is activated by CAF-derived growth factors and cytokines instead of androgen. Therefore, CAFs could be an important target to prevent androgen-independent outgrowth. 

In the clinical setting, serum PSA is the most useful biomarker to detect PCa. However, increased levels of serum PSA are also observed in cases of benign prostatic hyperplasia or inflammation of the prostate. PSA is a serine protease and member of the tissue kallikrein family of proteases and is produced in both normal luminal epithelial cells and well-differentiated PCa cells [43]. Transcription of the *PSA* gene is normally regulated by androgens through activation of AR signaling. In addition to androgens, PSA expression is induced through activation of AR signaling by CAF-derived growth factors and cytokines. In our laboratory, Sasaki et al. reported that fibroblasts directly affected PSA expression in LNCaP cells cocultured in vitro [17]. Among various CAF-derived growth factors and cytokines, we confirmed that EGF, IGF-1, and IL-6 directly increased PSA expression in LNCaP cells, suggesting that soluble factors derived from fibroblasts may function as AR-activating factors in the absence of androgen.

In our previous work, Sasaki et al. found that the PSA kinetics after ADT were not an accurate prognostic marker when considering serum PSA levels after ADT to determine the number of viable PCa cells [44,45]. Compared with rapid decreases in PSA after ADT, prolonged PSA decreases after ADT can predict favorable progression-free survival and overall survival. In this study, AR signaling in E9 cells, but not in F10 cells, was activated by paracrine signals derived from fibroblasts, suggesting that the androgen sensitivity of PCa cells may not reflect the AR dependency of PCa cells. Preservation of AR signaling after ADT may have an important role in maintaining the AR dependency of PCa cells. Thus, fibroblast-dependent AR activation after ADT may cause persistent activation of AR signaling in PCa cells, preventing loss of AR dependency after ADT. Notably, Sasaki et al. demonstrated that fibroblasts could enhance the treatment efficacy of ADT during in vivo tumorigenesis, resulting in a more favorable prognosis, e.g., prolonged serum PSA decline and maintenance of the efficacy of ADT [17]. Other researchers also demonstrated that normal human fibroblasts could inhibit the proliferation of tumor cells [46,47]. We still know very little about the tumor-promoting CAFs and the factors that distinguish CAFs from other fibroblasts found in the same tissue. Future studies are needed to identify the specific profiles of fibroblast-derived factors responsible for disease progression. Additionally, several AR variants (ARVs), derived from alternative splicing of the AR transcript, have been identified [48,49,50,51]. ARVs may emerge as more common mediators of androgen-independent and AR-dependent tumor progression, although their functions are still unclear. AR-V7 is a major splice variant expressed in human PCa that is associated with the development and progression of CRPC [52]. In human PCa cell lines, expression of AR-V7 mediates resistance to a new generation of AR-targeted therapies, such as enzalutamide and abiraterone [53]. In addition, Shimizu et al. recently reported that knockdown of AR-V7 in LNCaP95-DR cells did not restore sensitivity to docetaxel and cabazitaxel, suggesting that AR-V7 may be not involved in taxane resistance [54]. Thus, expression of AR-V7 has been proposed for the assessment of suitability for taxane chemotherapy [55]. 

With regard to the androgen sensitivity of PCa cells, our data showed the following results, in the presence or absence of fibroblasts: (1) tumor growth of E9 cells was significantly diminished after ADT compared with that in the sham group; (2) tumor growth of F10 cells was temporally reduced after ADT, but was restarted under androgen deprivation; and (3) tumor growth of AIDL cells was not decreased after ADT. These results established some important clinical concepts. For example, to treat certain PCa cells (e.g., E9 cells), fibroblast-target therapy should be avoided because of the preservation of AR signaling after ADT. Additionally, for certain PCa cells expressing AR-V7 (e.g., F10 cells), ADT may not be effective for treating CRPC. In these cells, the responsiveness to fibroblasts may not be associated with tumor growth, and the efficacy of ADT may be limited. Finally, in androgen-insensitive PCa cells (e.g., AIDL cells), ADT is completely useless because of the AR independence of PCa cells. 

Identification of improved, personalized treatments will be supported by recent major progress in the molecular characterization of early- and late-stage PCa. Indeed, such advancements have already led to novel classifications of prostate tumors based on gene expression profiles and mutation status and should greatly facilitate the choice of novel targeted therapies best tailored to the needs of patients [56,57], particularly for individual subgroups of patients, representing a major step towards personalized medicine adapted to the individual needs of patients with PCa. Selecting the optimal drug or drug sequence and combination for PCa treatment will be improved by the identification of molecular biomarkers predictive of response and progression. Similar to breast cancer, subdivisions of luminal A, luminal B, and basal subtypes, which exhibit different clinical prognoses and responses to ADT, have been proposed for PCa [58]. Such a classification may greatly support treatment choices for early- and late-stage disease and ultimately improve the overall survival rates and quality of life in patients with PCa. As we learn more about the genetic heterogeneity of PCa cells and mechanisms of treatment resistance, we expect that markers for treatment choice and response will be developed and validated to better guide treatment decisions [59,60].

Using three sublines of androgen-sensitive LNCaP cells, we demonstrated that loss of fibroblast-dependent AR activation in PCa cells (e.g., F10 cells) may be responsible for the development and progression of CRPC. In the absence of androgen, the AR dependence of PCa cells interacting with fibroblasts reflected the efficacy of ADT; for example, E9 cells could be controlled by ADT. To choose appropriate patients with advanced PCa for ADT, it is necessary to evaluate the degree of AR dependence in PCa cells interacting with fibroblasts before ADT is started. Further investigations are needed to identify clear molecular markers using biopsy tissue samples or bodily fluid samples derived from patients with advanced PCa. 

## 5. Conclusions

To prevent the development and progression of CRPC, the preservation of AR signaling after ADT is essential. In this study, we demonstrated that loss of fibroblast-dependent AR activation and expression of AR-V7 protein in PCa cells may be involved in the mechanism of acquired resistance to castration. In near future, clear molecular markers are needed to identify the degree of AR dependence in PCa cells interacting with fibroblasts before ADT is started, e.g., expression of AR-V7 proteins is one of the candidates.

## Supplementary Materials

The following are available online at https://www.mdpi.com/2077-0383/8/9/1379/s1, Figure S1: Effects of ADT on histopathological characteristics of xenografts derived from co-inoculation of E9 cells with fibroblasts in vivo; Figure S2: Effects of ADT on histopathological characteristics of xenografts derived from co-inoculation of F10 cells with fibroblasts in vivo; Figure S3: Effects of ADT on histopathological characteristics of xenografts derived from co-inoculation of AIDL cells with fibroblasts in vivo; Figure S4: Expression of EGFR protein in human PCa cell lines; Figure S5: Effects of growth factors on PSA secretion from LNCaP sublines in vitro; Table S1: MVD changes in E9 tumors after ADT; Table S2: MVD changes in F10 tumors after ADT; Table S3: MVD changes in AIDL tumors after ADT.
