# Supplementary material for: Loss of Fibroblast-Dependent Androgen Receptor Activation in Prostate Cancer Cells is Involved in the Mechanism of Acquired Resistance to Castration
Source: J Clin Med. 2019 Sep 3;8(9):1379. doi: 10.3390/jcm8091379 (PMC6780155; doi:10.3390/jcm8091379)
Supplement: Supplementary file 1 [file jcm-08-01379-s001.pdf]

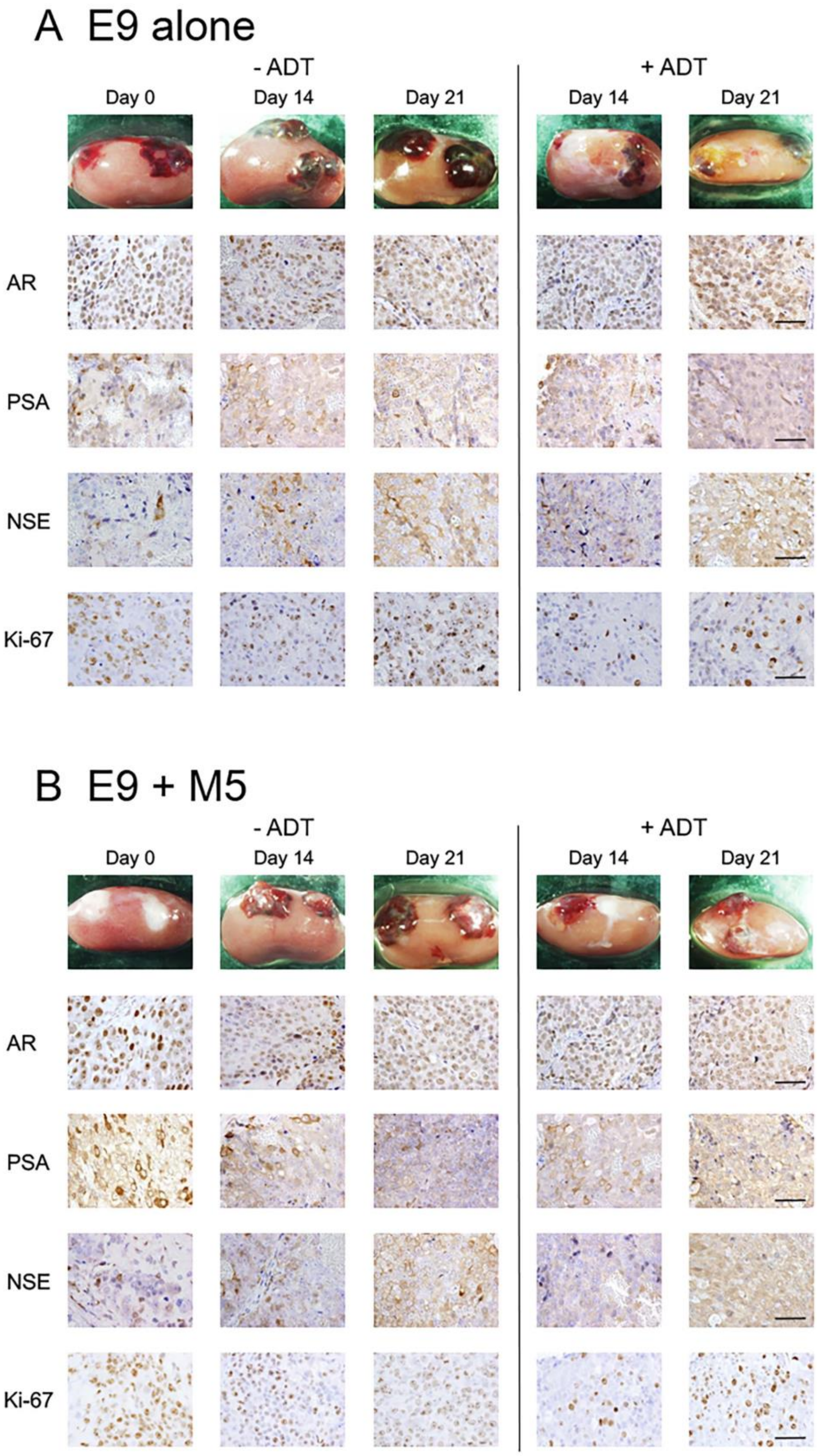

Figure S1. Effects of ADT on histopathological characteristics of xenografts derived from co-inoculation of E9 cells with fibroblasts in vivo. Characteristic gross appearances of E9 cells alone (**A**) and E9 cells plus pcPrF-M5 cells (**B**) in both untreated (sham-operated) and ADT-treated mice (bar = 2 mm). Representative images of AR, PSA, NSE, and Ki-67 staining of mice from each group on day 21 after ADT are also shown. Bar = 100 μm, magnification = 400×. ADT, androgen deprivation therapy; AR, androgen receptor; NSE, neuron-specific enolase; PSA, prostate-specific antigen; M5, pcPrF-M5.

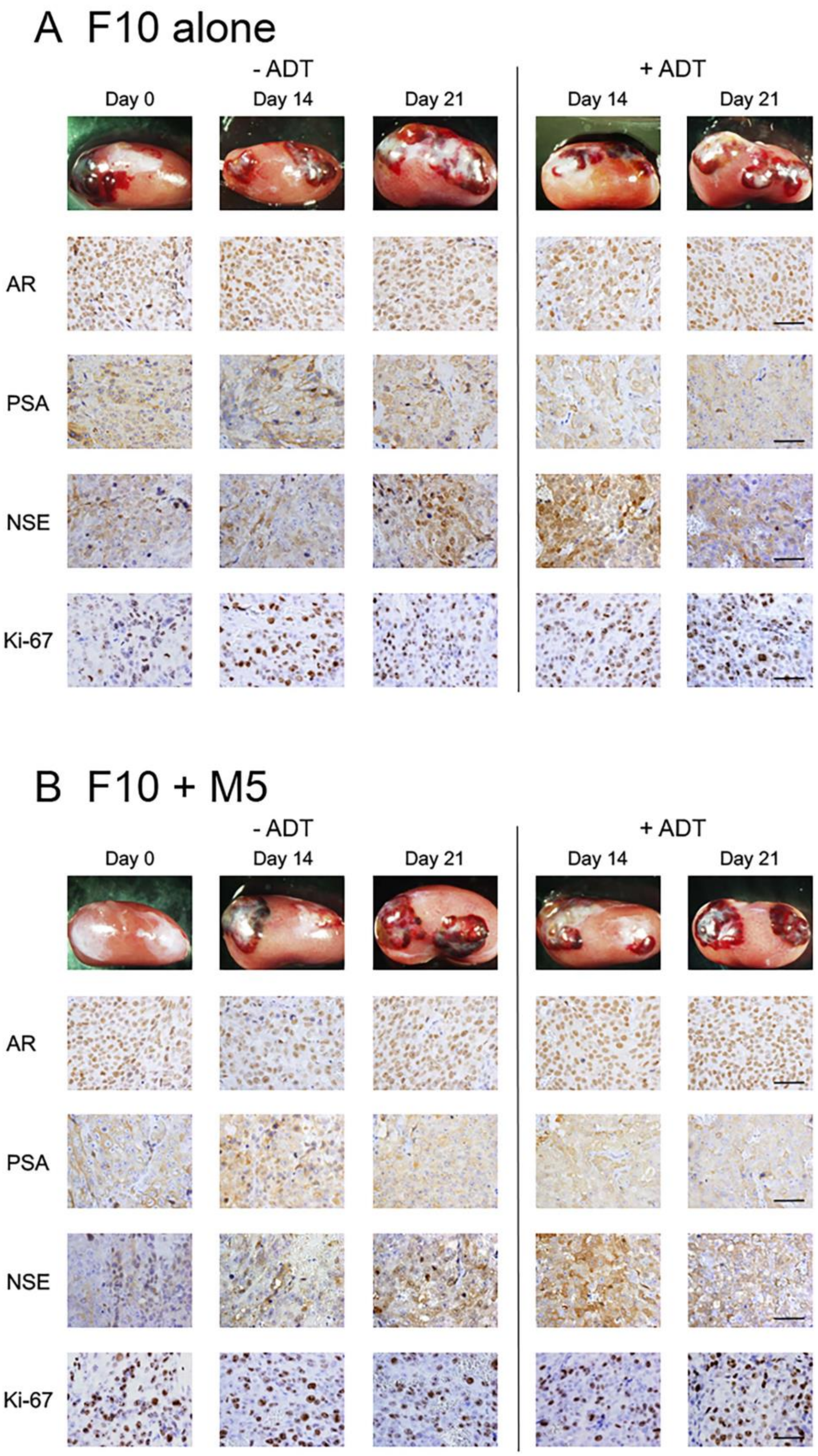

Figure S2. Effects of ADT on histopathological characteristics of xenografts derived from co-inoculation of F10 cells with fibroblasts in vivo. Characteristic gross appearances of F10 cells alone (**A**) and F10 cells plus pcPrF-M5 cells (**B**) in both untreated (sham-operated) and ADT-treated mice (bar = 2 mm). Representative images of AR, PSA, NSE, and Ki-67 staining of mice from each group on day 21 after ADT. Bar = 100 µm, magnification = 400×. ADT, androgen deprivation therapy; AR, androgen receptor; NSE, neuron-specific enolase; PSA, prostate-specific antigen; M5, pcPrF-M5.

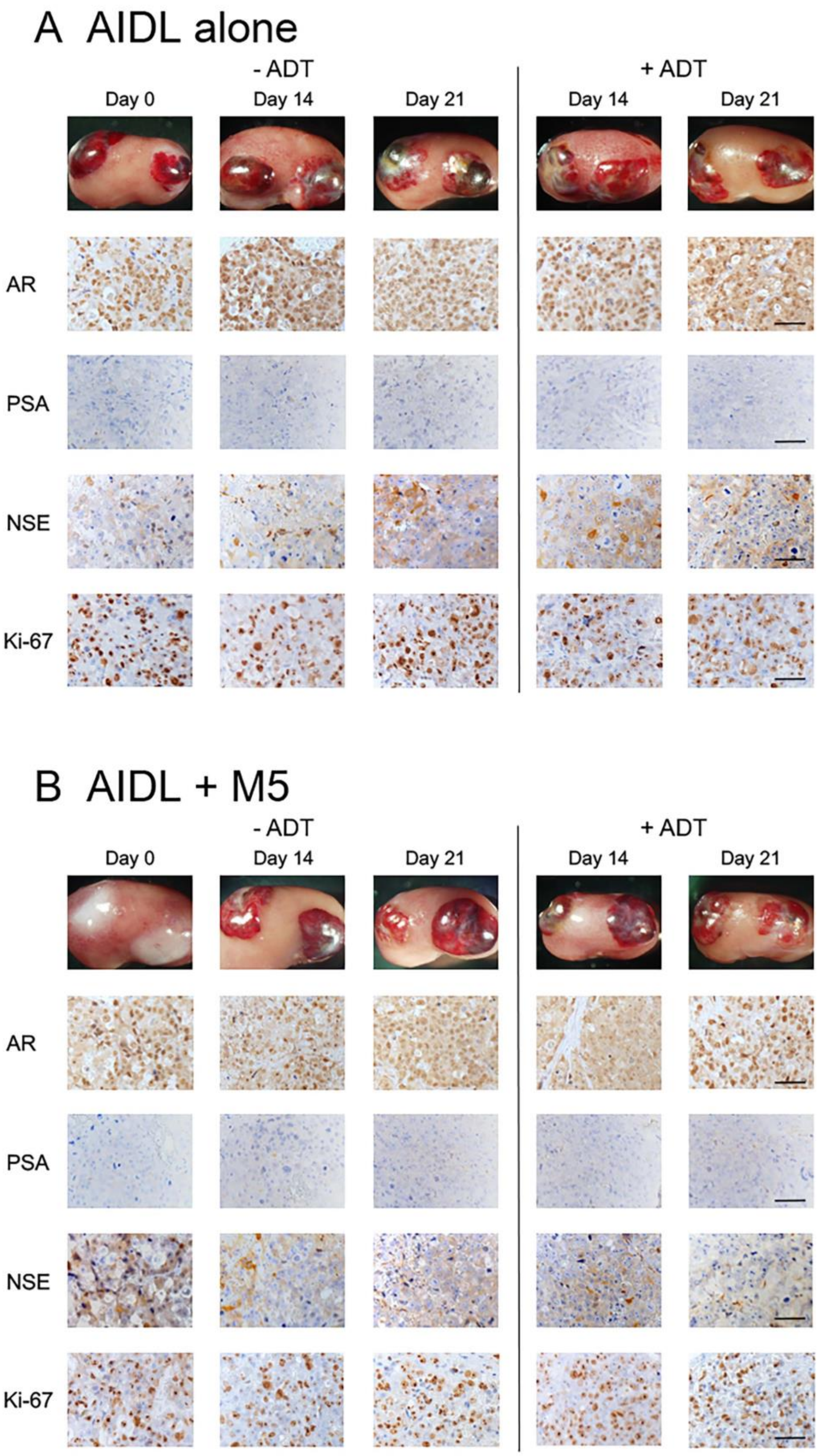

Figure S3. Effects of ADT on histopathological characteristics of xenografts derived from co-inoculation of AIDL cells with fibroblasts in vivo. Characteristic gross appearances of AIDL cells alone (**A**) and AIDL cells plus pcPrF-M5 cells (**B**) in both untreated (sham-operated) and ADT-treated mice (bar = 2 mm). Representative images of AR, PSA, NSE, and Ki-67 staining of mice from each group on day 21 after ADT. Bar = 100 µm, magnification = 400×. ADT, androgen deprivation therapy; AR, androgen receptor; NSE, neuron-specific enolase; PSA, prostate-specific antigen; M5, pcPrF-M5.

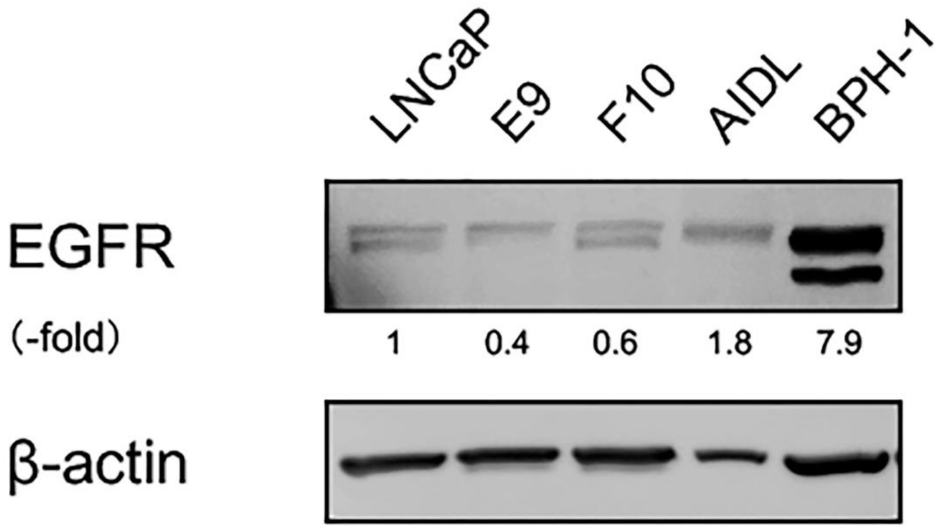

Figure S4. Expression of EGFR protein in human PCa cell lines. Cell lysates from growing cultures of parental LNCaP cells, LNCaP sublines (E9, F10, and AIDL cells), and BPH-1 cells were subjected to western blotting and probed with antibodies against each protein. Protein levels were compared using actin as a loading control. BPH-1 cells were used as a positive control for detection of EGFR protein. EGFR, EGF receptor.

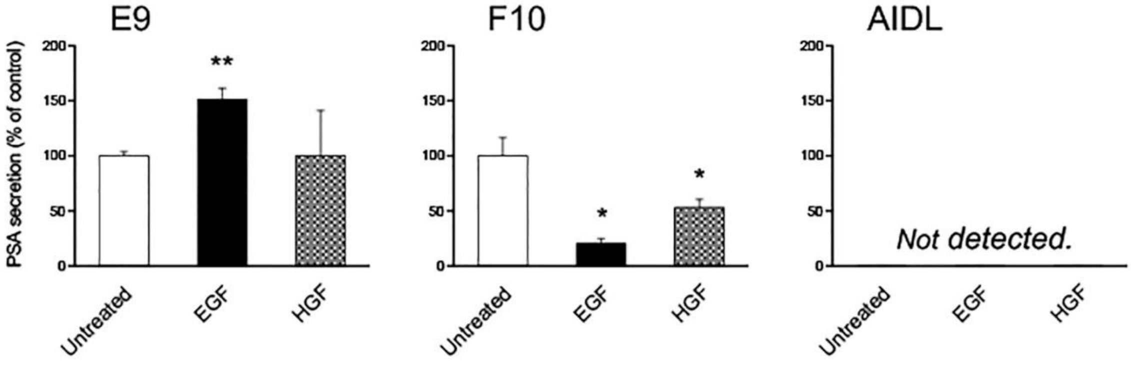

Figure S5. Effects of growth factors on PSA secretion from LNCaP sublines in vitro. LNCaP sublines were treated with 10 ng/mL of EGF or HGF for 4 days in phenol red (-) RPMI-1640 with 1% CS-FBS containing DHT (0.1 nM). For quantitation of PSA, aliquots of conditional medium were subjected to ELISA. * $P < 0.05$, ** $P < 0.01$ versus untreated control. PSA, prostate-specific antigen; DHT, dihydrotestosterone.

Table S1. MVD changes in E9 tumors after ADT.

Days after ADT	MVD			
	E9 alone		E9 + M5	
	Sham	ADT	Sham	ADT
0	3.9 ± 1.2		4.3 ± 0.8	
14	4.2 ± 0.9	3.5 ± 0.9	4.8 ± 0.9	3.5 ± 0.9
21	4.7 ± 1.1	3.5 ± 1.3	5.2 ± 0.9	4.8 ± 1.2

ADT, androgen deprivation therapy; MVD, microvessel density; M5, pcPrF-M5.

Table S2. MVD changes in F10 tumors after ADT.

Days after ADT	MVD			
	F10 alone		F10 + M5	
	Sham	ADT	Sham	ADT
0	8.0 ± 2.3		7.6 ± 1.9	
14	8.6 ± 1.6	8.4 ± 2.1	8.7 ± 2.8	8.6 ± 1.6
21	8.8 ± 2.1	9.9 ± 1.7	8.8 ± 1.6	8.8 ± 2.6

ADT, androgen deprivation therapy; MVD, microvessel density; M5, pcPrF-M5.

Table S3. MVD changes in AIDL tumors after ADT.

Days after ADT	MVD			
	AIDL alone		AIDL + M5	
	Sham	ADT	Sham	ADT
0	16.0 ± 2.2		15.4 ± 1.9	
14	16.0 ± 2.2	15.9 ± 2.5	17.0 ± 2.9	16.2 ± 2.3
21	16.2 ± 3.2	16.6 ± 3.2	16.1 ± 3.2	16.9 ± 1.8

ADT, androgen deprivation therapy; MVD, microvessel density; M5, pcPrF-M5.
